# Feasibility of a Pharmabuddy Care Service for patients with Parkinson’s disease

**DOI:** 10.1186/s12913-024-12057-x

**Published:** 2024-12-18

**Authors:** C. C. M. Stuijt, F. Karapinar-Çarkit, C. van de Steeg – van Gompel, T. van Laar, B. J. F. van den Bemt, M. Heringa

**Affiliations:** 1 Center of Excellence On Parkinson’s Disease [Punt Voor Parkinson], Schaaksport 100, Groningen, the Netherlands; 2 Department of Clinical Pharmacy & Toxicology, Maastricht University Medical Centre+, Maastricht, the Netherlands; 3 CARIM School for Cardiovascular Disease, Maastricht University, Maastricht, the Netherlands; 4 SIR Institute for Pharmacy Practice and Policy, Leiden, the Netherlands; 5 Present Address: Department of Neurology, University of Groningen, University Medical Center Groningen, Groningen, the Netherlands; 6 Department of Pharmacy, Sint Maartenskliniek, Nijmegen, the Netherlands; 7 Department of Pharmacy, Radboud University Medical Centre, Nijmegen, the Netherlands

**Keywords:** Specialized pharmaceutical care, Parkinson's disease, Implementation & education

## Abstract

**Background:**

Pharmaceutical care for patients with Parkinson’s disease (PD) is complex. Specialized pharmaceutical care provided by a dedicated pharmacy team member (pharmabuddy) for these patients may reduce medication-related problems. The feasibility of this service for PD patients is unknown.

**Objective:**

To evaluate the feasibility of a Pharmabuddy Care Service (PCS) for PD patients in primary care pharmacies.

**Methods:**

Pharmabuddies who offered PCS were invited to fill in a questionnaire to evaluate the feasibility of PCS. Patients received a patient questionnaire and were invited for an interview. Patient records provided information on medication-related problems and interventions. Feasibility was evaluated conform four domains of Bowen’s Framework. First, *acceptability* included patients’ satisfaction ratings and experiences, Pharmabuddy impression on start and continuation of the service. Second, *demand* included use by patients and provision by pharmabuddies. Third, *implementation/practicality:* implementation indicators and barriers and facilitators from patient- and pharmabuddy perspective and fourth *limited efficacy:* effect of the PCS on PD symptoms, medication related problems identified and interventions from patient records.

**Results:**

Twenty-three (59%) patients completed the questionnaire, 9 were interviewed, 12 (67%) pharmabuddies responded. *Acceptability* was high among patients (mean 9.5 (SD 1.3) out of 10), 6 (50%) pharmacies (still) provided PCS. D*emand*: 56% of patients had 1–2 contact moments, 28% two or more. Ten pharmacies provided up to 14 patients per pharmacy with PCS, one up to 24. *Implementation/practicality*: important barriers were time constraints and perception of other healthcare professionals. Positive reactions from patients encouraged pharmabuddies to carry out their PCS-activities. Patients were positive about pharmabuddy’s listening competency while knowledge could be improved. *Limited efficacy:* from patient records, 89 interventions were made in response to 93 (median 3 per patient (range 1–16)) medication related problems or questions, with 20 (87%) patients reporting a positive effect from their interaction with a pharmabuddy.

**Conclusion:**

This study shows PCS is highly appreciated by responding patients and can be feasible for primary care pharmacies. Several implementation issues are still present. Future studies should focus on quantifiable effects of PCS services as well as resource and perception hurdles.

**Supplementary Information:**

The online version contains supplementary material available at 10.1186/s12913-024-12057-x.

## Background

Parkinson’s disease (PD) is the fastest growing neurological disorder in the world. The prevalence of PD more than doubled from 2,5 million patients in 1990 to over 6 million in 2016 and will be doubled again in the forthcoming 30 years. Combined with its considerable burden of illness, enormous challenges regarding access to care in both primary care and neurology services are faced [1]. The treatment of PD is currently mainly focused on symptomatic relief by drug therapy. However, effective management of PD is complex and treatment choices are a continuous balance between effectiveness and adverse drug events (ADEs) [2, 3]. With progression of the disease, neuropsychiatric and autonomic symptoms as well as motor complications become more prominent, leading to complex medication regimens [4]. PD patients have 3 times more comorbidities and use consequently 30% higher numbers of medication as compared to patients without PD [5]. This increasing pill burden generates difficulties for patients to handle medication correctly and increases even more risk on ADEs [6].

Medication has a prominent role in the treatment of PD. Therefore, pharmacists should be an integral part of the PD health care team(7,8). Pharmacy services have been shown to improve medication treatment in PD patients by reduction of number of intakes and improvement of adherence as well as quality of life [7, 8]. Pharmabuddy Care Service (PCS) is one of the most recent developments in this field in which pharmaceutical care has evolved into close connection with patients. PCS is based on a specialized and dedicated pharmacy team member (the pharmabuddy) who is assigned to a particular patient. Recent data on the PCS concept for palliative patients, showed a decrease of pharmaceutical problems, improved collaboration with other healthcare professionals (HCPs) and diminished waste of medication which resulted in improved satisfaction of patients and their caregivers [9]. This person-centred approach, focused on a patient’s needs and health status, seems to be promising in management of patient and medication safety in specific high risk, relatively small patient groups, as compared to current pharmaceutical care. Therefore, PCS education on Parkinson’s disease has been developed and deployed for pharmacy teams in the Netherlands [10]. However, the feasibility of PCS has never been studied, which is a prerequisite before implementing this on a larger scale. Therefore, we analysed the feasibility of PCS for PD patients in primary care pharmacies.

## Methods

### Design and setting

This mixed-methods study was based on Bowen's Framework for feasibility studies [11]. This framework has been used to analyse feasibility of various public health and preventive medicine interventions [12, 13]. Although Bowen’s framework encompasses eight domains, five domains were applicable to assess the feasibility of PCS for PD patients at this stage, being *acceptability, demand, implementation, practicality* and *limited-efficacy*. The remaining three domains *adaptation (*change of program content*)*, *integration (*need for system changes*)* and *expansion (*already successful intervention in a different area*)* were not applicable, because PCS is not yet disseminated. Based on the five applicable domains, 12 outcomes and 22 sub-outcomes were defined from a patient—and pharmabuddy perspective (see Table 1).

**Table 1:** Feasibility domains, outcomes and sub-outcomes for Pharmabuddy Care Service on Parkinson’s disease from patient and pharmabuddy perspective

Domain	Perspective	Outcome	Sub-outcome	Data source
1. **Acceptability** *“how individuals involved react to the intervention”*	Patient	1.1 Patient satisfaction and appreciation of PCS	1.1.1 satisfaction rating (grading of PCS)	Patient questionnaire
			1.1.2 PCS experience (e.g. on communication and knowledge of pharmabuddies)	Patient questionnaire (Likert-scale questions) and interview
	Pharmabuddy	1.2 situation around PCS in pharmacy	1.2.1 Pharmabuddy impression on introduction and continuation of the service	Pharmabuddy questionnaire
**2. Demand** *“Likelihood of the program to be used”*	Patient	2. 1 Actual use	2.1.1 Contact frequency in the last 6 months	Patient questionnaire
			2.1.2 N contacts initiated by patient	Patient records
		2.2 Anticipated use	2.2.1 expressed interest or intention to use	Patient interview
	Pharmabuddy	2.3 Actual PCS provision	2.3.1 N patients in PCS per pharmacy 2.3.2 N PCS providing pharmacies	Pharmabuddy questionnaire
			2.3.3 N active pharmabuddies per pharmacy	
		2.4 Referrals	2.4.1 N referrals by other HCPs	Pharmabuddy questionnaire
**3. Implementation/** **practicality** *“Degree, success or failure of execution* *factors affecting implementation”* ”*extent of delivery in situation constrains”*	Patient	3.1 Tips and tops	3.1.1 Facilitators and barriers/ tips and tops for implementation	Patient questionnaire and interview
	Pharmabuddy	3.2 Indicators of implementation	3.2.1 Use of provided materials	Pharmabuddy questionnaire
			3.2.2 Extent of implementation effort	
			3.2.3 Lag time between course completion and intervention initiation	
			3.2.4 Stakeholder involvement (N of referrals)	
		3.3 Barriers and facilitators	3.3.1 Facilitators and barriers for implementation: pharmabuddies (e.g. presence of necessary knowledge)	Pharmabuddy questionnaire
		3.4 Conditions for PCS	3.4.1 ways (including ease/constraints) of patient identification	Pharmabuddy questionnaire
			3.4.2 N pharmacies started inviting patients: continuation ability	
**4. Limited efficacy** **“***effect on medication use, knowledge of disease, complaints”*	Patient Pharmabuddy	4.1 Clinical outcomes	4.1.1 Perceived benefits/deterioration by patients	Patient questionnaire (open-ended questions) and interview
			4.1.2 Effect on PD symptoms	Patient questionnaire (open-ended questions) and interview
		4.2 medication-related problems + interventions	4.2.1 N and type of medication-related problems identified by Pharmabuddy’s	Patient records
			4.2.2 N and type of interventions performed by Pharmabuddy’s	Patient records

*HCP* Health Care Professional, *PCS* Pharmabuddy Care Service, *PD* Parkinson’s Disease, *N* number

The domains *implementation* (implementation of PCS as planned and proposed) and *practicality* (constraints like resources, commitment or time preventing delivery of PCS) were interlinked and therefore evaluated together.

From 2018—2020 pharmacy teams were invited via a national open registration for PCS education on Parkinson’s disease and participation in the PCS. The 18 teams which started PCS education and PCS services in 2018 and 2019 were invited to collect data for this feasibility study on both patient- and pharmabuddy sources, existing of (i) patient questionnaires, (ii) patient interviews, (iii) patient records and (iv) pharmabuddy questionnaires.

The pharmacy teams which started the PCS education in 2020, were invited to collect information from patients (and not from pharmabuddies) until mid-2022 (including two lockdown periods of COVID-19 pandemic). Each participating pharmacy deputed one pharmacist and one or two pharmacy technicians to the PCS education course.

### Intervention: Pharmabuddy Care Service (PCS) program

The core component of PCS is coupling pharmabuddies to individual PD patients and their caregivers and HCPs. PCS is initiated by the pharmabuddy having a conversation with the patient and the caregiver (if available), to build a relationship based on trust, to identify the needs, concerns and goals connected with pharmacotherapy and their disease. After this initial consultation pharmabuddies regularly (e.g. linked to the 3-monthly dispensing of medication) or pro-actively contact the patient/caregiver to check on potential medication- or condition related problems (e.g. difficulties with medication intake; new non-neurological prescriptions potentially interfering with PD treatment) and contact other involved HCPs if needed. Pharmabuddies encourage patient initiated contact and anticipate on potential problems by checking on triggers like (risk on) falls, nausea and recent unplanned hospital admissions. In addition, they coordinate dispensing, including support on the logistics of medication (e.g. synchronise dispensing of all chronic medicines, sending emails or text message to patients to support medication ordering) to unburden the patient. Both, pharmacists and technicians, can act as pharmabuddies, although generally a duo of pharmacy technicians had this role, with pharmacists as back-up. Pharmacy technicians have an important executive role in the Netherlands and are trained and skilled to have face-to-face interaction with patients on their medication [14]. All participating pharmacy teams received a 3-day course on Parkinson’s disease, with focus on pathophysiology and pharmacological treatment including (practical) knowledge, communication and organization of PCS (Appendix 1). All teams were provided with checklists to support the implementation of PCS in their daily routine.

### Data sources and- analysis

Information on (sub-)outcomes and a brief explanation per domain connected with the data-sources is provided in Table 1.

#### Patient questionnaires

Pharmabuddies were asked to invite all patients who agreed to receive PCS and had been contacted at least once by a Pharmabuddy to complete a questionnaire [15, 16]. A 15-item patient questionnaire covering subdomains from all 5 Bowen’s domains was drafted, focusing on patient experiences with the pharmabuddy, knowledge of the pharmabuddy, topics discussed, and the perceived change in clinical signs (Appendix 2).

The questionnaire was available on paper and electronically in Survalyzer. The questionnaire was tested by a PD patient on comprehensibility and time to complete, not leading to major changes.

Data from the patient questionnaires were analysed in SPSS 28. The multiple choice questions were analysed with descriptive statistics. Open-ended questions, were categorized per outcome in duplo.

#### Patient interviews

Patients were invited for an interview via the last question in the patient questionnaire. Patient interviews were designed to obtain more in-depth information on all five Bowen’s domains, focusing on how patients experienced PCS, especially regarding the intentions to use the program and patients’ needs. A topic guide for the interviews was developed to cover these domains (Appendix 3a) [17]. The semi-structured interviews were conducted by either a senior pharmacist-investigator or a trained master student pharmacy by phone from May to June 2020. All interviews were audio recorded and transcribed verbatim. Data from patient interviews were transcribed and content analysis was performed in NVivo12. First, all interviews were coded with open codes by two authors (CS and MH), whereas possible differences in the coding were discussed until consensus was reached. Second, codes were clustered into topics within the predefined sub-outcomes. Interviews were performed until a feasible sample was reached and little new information came in to address the research question.

#### Patient records

The pharmacy teams provided information taken from patient records, which was extracted from pharmacy information systems in order to assess limited efficacy. The length of PCS activities ranged from a few years to just one intake. Pharmacy information systems in the Netherlands contain, apart from medication dispensing histories for each patient, free text related to contacts between pharmabuddies and patients. Topics discussed included drug -drug interactions, logistic- and medication-intake issues (Appendix 3b). It is possible to upload documents e.g. PCS intake forms (with information on patient preferences, wishes), for extra background information. Data on pharmaceutical care provided was put in an Access database of contact moments. Medication-related problems were classified based on the Systematic Tool to Reduce Inappropriate Prescribing classification, with addition of logistic and administrative items [18].

#### Pharmabuddy questionnaires

The questionnaire for pharmabuddies focused on Bowen’s domains *acceptability, demand, implementation/practicality*. Questions included the numbers of pharmabuddies active in the pharmacy, numbers of PD patients contacted, patient selection, if and how other HCPs were informed about PCS, implementation and practicality issues (facilitators, barriers) as well as activities around the PCS program (Appendix 4). Questions were based on a previous report on a program for palliative care with pharmabuddies [19]. Pharmabuddy questionnaires were sent electronically by Survalyzer to all pharmabuddies of the 18 pharmacies educated first. Automated reminders were sent after 2 weeks in order to increase the response rate. Questionnaires were filled out by pharmabuddies at the beginning of 2020.

Multiple choice questions were analysed with descriptive statistics. The content of open-ended questions was categorized per (sub) outcome in duplo.

#### Ethics and confidentiality

The Medical Ethics Review Board of the University Medical Centre Utrecht concluded that this study did not require WMO approval, because it is not about clinical research with human participants as intended by the Dutch Medical Research Involving Human Subjects Act. Therefore, Human Ethics and Consent to Participate declarations is not applicable. Patients gave informed consent for the interviews and they were also asked to consent the recording of the interviews. Questionnaires and patient records were anonymized and stored on protected servers.

## Results

In total 57 pharmabuddies (30 pharmacy technicians, 27 pharmacists) from 27 pharmacies were educated in 2018 -2020. Ten pharmacies (37%) were located in two northern provinces (Groningen, Friesland) and another 10 pharmacies were in the western part of the Netherlands (region Leiden), whereas the other seven pharmacies (26%) were spread across the country. Two of these pharmacies were located in hospitals, serving outpatient clinics.

Nine pharmacies of the 27 educated pharmacies (33%) invited 39 patients in total for the patient questionnaire, of which 23 (59%) completed the questionnaire and 9 (39%) were interviewed. Two-third (67%) of interviewed patients (9) were female, eight had spouses (89%) and 2 PD patients had an advanced therapy (Deep Brain Stimulation, Duodopa pump). All interviewed patients handled their medication independently.

Twelve pharmabuddies of the first cohort (2018–2019) educated pharmacies completed the pharmabuddy questionnaire (67% of 18 invited), five pharmacies provided information on patient records.

### Acceptability

Most patients indicated in the patient questionnaire that they would recommend PCS to other patients (rating 9.5 out of 10, SD 1.3 ( outcome Appendix Table 1). No patients made a negative statements.

The positive attitude and experiences of patients towards PCS was confirmed both by the questionnaires and interviews. In the questionnaires patients rewarded especially the listening, advices for personal situation, accessibility (possibility to ask questions), PD-treatment knowledge and interest in the personal situation (Table 2), which was confirmed by the interviews (Table 3, Quote 2–5).

**Table 2:** Experience of patients in pharmabuddy contact (*n* = 23 patient questionnaires)

	**Applicability**		
**Topic**	**Always/Often, n (%)**	**Sometimes/** **Never, n (%)**	**NA, n (%)**
Listening well	21 (91)	-	2 (9)
Possibility to ask questions	20 (87)	1 (4)	2 (9)
Interested in personal situation	16 (70)	3 (13)	4 (17)
Advice for personal situation	17 (74)	2 (9)	4 (17)
Good explanation	20 (87)	1 (4)	2 (9)
Knowledge on PD treatment	18 (78)	2(9)	3 (13)
Addition on top of other HCPs	15 (66)	4 (17)	4 (17)
Brand switch	8 (35)	1 (4)	14 (61)

*NA* not applicable

**Table 3:** Quotes from patient interviews per domain and sub-outcome

domain	Out-come	interviewee	Quote number	Quote
acceptability	Patient satisfaction & experience with PCS	**Accessibility**		
		P1	Q1	‘*I have the feeling she [the pharmabuddy] has more time, space for me. The neurologist will be disturbed by me, specifically now during Corona. I am unsure about myself, I have the feeling to be too much so I’m looking for reassurance. I had the idea that I had to stop medication but maybe it wasn’t a good idea. One becomes unsure and then it is convenient to have an accessible back-up.’*
		**Listening ear without time constraints**		
		P2	Q2	*‘They [the pharmabuddies] have more time, per contact moment.’ Advices on food and effect on medication effectiveness that kind of things. She really took me seriously and listened carefully.’*
		P3	Q3	*‘the pharmacist visits me at home, if I call the pharmacy they visit me very shortly afterwards. I am very satisfied.’*
		P2	Q4	*‘She understands me for example with medication switches which is really annoying. She has more knowledge so she understands better my situation e.g. when I tell her that I’m tired which is correlated with Parkinson’s. That I have to take a nap in the afternoon otherwise I can’t stand it’*
		**Direct contact with skilled person**		
		P4	Q5	*‘I really appreciate the direct contact with the pharmabuddy, a pharmacy technician needs always consultation of a pharmacist. A direct conversation on different topics, I really appreciate that.’*
demand	expressed interest or intention to use	**Contact frequency**		
		P4	Q6	*‘It [the contact with someone from the pharmacy] has been quite frequent lately. After trademark switches: 3 – 4 times the new pharmabuddy and two time with the coordinator, to talk this person.’*
		**Initiative contact (buddy vs patient)**		
		P5	Q7	*‘There has been a couple of times contact, it was initiated by the pharmabuddy’*
		P6	Q8a	*‘I initiated the contact myself: wanted to have information on Retard preparations.’*
		P6	Q8b	*‘Actually I would rather have no appointment if I don’t need it. Let’s put it this way: I have sufficient people to talk to. But if it is possible to make an appointment in short notice, I would very much appreciate.’*
		**Pharmabuddy is the first person to be contacted (yes and no)**		
		P7	Q9a	*‘It’s a positive development. Since January this year she is my buddy, I ‘m very positive on it. I know where to find her and *vice versa*, so that’s positive. ‘*
		P7	Q9b	*Interviewer: have you ever experienced adverse drug reactions? ‘Yes, for sure.’ Interviewer: Can you give examples? ‘Fatigue, spongy head, malaise, urge complaints.’* *Interviewer: have you ever discussed these complaints with someone? ‘Yes with the specialized Parkinson nurse and recently also with a pharmacy technician who became my buddy. But it is new for her and she still has to learn. ‘*
		P4	Q10	*‘I noticed the neurologist is better in which medication to be added on top of the combination I already use, specifically in case I have questions. ‘* *Interviewer: so you would go to the pharmacy for less complicated things and for solutions with regard to content the neurologist?’That’s correct.’*
		P8	Q11	*‘Yes, we have had contact: she called me yesterday about the medicine. I think that is nice. For the rest we will lean on the prescription of the GP, finally the GP is the first contact person. I will contact the GP as the expert [on medication]. ‘*
Implementation/practicallity	tips and tops for implementation	**Education by the patient**		
		P9	Q12a	*Interviewer: if I understand you correctly: you help the pharmabuddy by informing her about good care? ‘More from both sides I think: we inform each other.’*
		P4	Q12b	*‘It was a good conversation. I noticed she could still learn something from me. That’s no problem, it is from the clinical practice. ‘*
		**Tip discretion**		
		P7	Q13	*‘… while entering the crowded pharmacy I can show my pharmabuddy card and will receive a separate treatment. That’s rather embarrassing. However, discretion is guaranteed by taking me to a separate room.’*
		**Tip&top material**		
		P1	Q14	*‘A business card is a very positive thing: it states when and how to get in contact with the pharmabuddy*.’
		**Tip&top patient preparation**		
		P1	Q15	*‘For preparation of a phone call it would be very positive to receive an email. So that I will write things down. Or put the business card on the note board over here. That makes things easier. It is really a solution!’*
Limited efficay	Perceived benefit or deterioration	**Medication intake/organization**		
		P5	Q16	*‘Medication can be ordered directly *via* the pharmacy instead of the GP, just call or send an email to the buddy and medication is delivered at home. ‘*
		P5	Q17	*‘Periodically information on food and what should be done [in combination with medication]. My husband and I are very happy with that. ‘*
		**General monitoring**		
		P8	Q18	*‘The pharmacist has been visiting us at home to verify on all medications in use. I liked it that she keeps an eye on it.’*

*Q* quote

The interviews identified 4 patient satisfaction related-topics: 1. increased accessibility of Parkinson care (Table 3, Quote 1) 2. attention without time constraints (Table 3, Quote 2) 3. personal approach and flexibility of pharmabuddies (Table 3, Quote 3) 4. direct contact with skilled persons (Table 3, Quote 4,5). Accessibility included literally ‘the shop around the corner’ and also a more approachable HCP, compared to the neurologist (Table 3, Quote 1).

After implementation of PCS in the pharmacy, pharmabuddy questionnaires showed that 50% of the 12 responding pharmacies were still active with PCS, after having finished the training. The others had stopped PCS activities or had had no patient inclusion in the program (outcome Appendix Table 1).

### Demand

The patient questionnaires showed that PCS consisted of a variable amount of contacts per patient with pharmabuddies in the preceding 6 months, varying from just one contact (42%) to > 3 contacts (28%) (outcome Appendix Table 1). From patient interviews three topics on demand (expressed interest or intention to use) were extracted: (1) contact frequency, (2) initiation (patient or pharmabuddy) of the contact and (3) pharmabuddy as the first HCP to be contacted. Interviewed patients had one to five contact moments (Table 3, Quote 6). Contact with the pharmabuddy was both by initiation of the patient and the pharmabuddy although not all patients needed an extra HCP (Table 3, Quote 7–8). Despite positive experiences and high satisfaction, some interviewed patients indicated to feel more confident with other HCPs (f.i. specialized Parkinson nurses or neurologists) compared to the pharmabuddy, especially in case of more complex decisions (Table 3, Quotes 9b,10). However, specific practical medication-related issues (e.g. intake with food, problems with blister packaging) were discussed with a pharmabuddy, also because of the good accessibility of the pharmabuddy (Table 3, Quote, 8b). Generally, combinations of medication or dosage changes were discussed with their neurologist or GP (Table 3, Quotes,10,11).

The pharmabuddy questionnaires showed that half of participating pharmacies provided for ≤ 5 PD patients PCS, whereas others provided pharmabuddy care for 10 patients (between 1–24). These contact moments were generally provided and initiated by 1–2 pharmabuddies. Patients were referred to pharmabuddies by other HCPs in 6 pharmacies (50%), mostly by PD nurses (outcome Appendix Table 1).

### Implementation/practicality

Patients expressed inadequate knowledge of pharmabuddies in interviews, although pharmabuddies had confidence in their knowledge and skills. Patients noticed that pharmabuddies could learn from their experiences, and advised the pharmabuddies to do so. Other tips from patients included practicalities like sending emails to order medication, business cards with buddy information or more private entrances to the pharmacy (Table 3, Quote 12a,b, 13–15).

Six pharmacies (50%) of the pharmacies who completed the pharmabuddy questionnaire were still active with PCS (outcome Appendix Table 1). The majority of pharmacies were satisfied with the efforts of pharmabuddies on implementing PCS and used several supportive materials. Several facilitators and barriers on implementation of PCS were mentioned in the pharmabuddy questionnaire. Positive patients and HCPs reactions were facilitators. Time constraints was the most important barrier. Pharmabuddies retrospectively declared that more HCPs should have been informed about the introduction of PCS, the content of the program and potential benefits for PD patients. One citation stated: *‘Doctors and nurses had to be informed very thoroughly, they were rather sceptic about the project’* (outcome Appendix Table 1).

### Limited efficacy

The patient questionnaires showed that 20 patients (87%) had a positive overall benefit of the contacts with a pharmabuddy, which was also reported by interviewed patients (outcome Appendix Table 1). Two topics on limited efficacy could be extracted from the patient interviews: (1) medication intake and—organisation and (2) general monitoring (overview on all medications used, in conjunction with the patient). The interviewed patients were positive about the coordination of dispensing medication of the pharmabuddies, including support to order medication with periodic information on medication intake. Also the reassurance on medication handling was appreciated (outcome Appendix Table 1) (Table 3, Quote 1 and 16–18).

Based on the patient records of 21 patients in five pharmacies, a median of 3 drug related problems/questions (ranging from 1–16) were discussed per patient over a variable time period from a few years to just one intake. The majority (77%) was related to medical or pharmaceutical issues, like adverse drug reactions, PD complaints, evaluation of previous interventions or adherence/ medication intake problems and to switching between medication brands. Other topics included logistic problems (Table 4). 29 interventions (33%) were on pharmacotherapeutic changes/adjustments during consultations, while 33 (37%) was on information, advices and support for patients (Table 4). Pharmacotherapeutic interventions were accepted in 50% of the cases, others were not stated f.i because it was planned to be discussed during the next consultation with neurologist or specialised nurse.

**Table 4:** Problems and interventions per type and sub-type amongst PD patients participating in PCS (*n* = 21)

problem (*n* = 93)	Type	Subtype	number	
	**Clinical (medical/pharmaceutical)**		**72**	
		Evaluation (of previous interventions)		19
		Complaints (not directly medication-related)		15
		Parkinson complaints (potential non-optimal treatment)		14
		Adherence problems / medication intake problems		13
		(potential) adverse drug reaction		8
		Other		3
	**Administrative (prescription, reimbursement, dosage)**		**11**	
	**Logistics (supply or dispensing problems)**		**10**	
**Interventions (*n* = 89)**	**Type**	**Subtype**	**number**	
	**Pharmacotherapeutics**		**29**	
		(advice for) start on medication		9
		(advice for) dosage change		9
		(advice for) substitution		2
		(advice for) stop medication		2
		Advices on medication use		7
	**Consult**		**33**	
		Information/support		17
		Referral to other HCP		10
		Consultation of other HCP		6
	**Administrative**		**16**	
		Adjustment of medical record		13
		Prescription explanation		2
		Delivery of actual medication overview		1
	**Logistics**		**11**	
		Start automatic repeat prescription delivery or unit dose packaging (UDP)		5
		Changes in numbers of medications or content of UDP		4
		Dispensing of different (packaging) form		2

## Discussion

This is the first study on the feasibility of implementation and acceptability of Pharmabuddy Care Service (PCS) for a PD population at high risk for medication-related problems. Only one study reported data on the feasibility of pharmacist interventions in a PD population, however, in combination with speech therapy and physiotherapy, and via telehealth care, so describing a different approach (23). Proper service of a pharmacist/pharmabuddy in the Netherlands is even more important as PD patients in the Netherlands generally visit their neurologist only 2–4 times a year. Furthermore, they do not consult their GPs, due to the fact that patients feel that GPs lack expert knowledge and skills on PD [20].

Our study clearly showed that patients are very satisfied with PCS and resulted in a reduction of complaints and questions about medication as well as less concerns. The trained pharmacy technicians and pharmacists were able and willing to fulfil the ambition of PCS, creating a patient- centred approach for PD patients. However, implementation and continuation of PCS was difficult. Fifty percent of the responding pharmacies became active and invited patients, which might be related to the fact that PCS activities were not reimbursed. Pharmabuddies also experienced obstacles to convince other HCPs of the added value of PCS. This might be due to the fact that doctors and nurses generally have limited experience in collaborating with pharmacists on PD patients, therefore not knowing what they are able to contribute [21]. On the other hand, PD patients from six pharmacies were referred to pharmabuddies by HCPs after the introduction of PCS, which would not have occurred before the introduction of PCS.

Unfortunately, patients experienced insufficient knowledge and skills of pharmabuddies on Parkinson’s disease and its treatment. This might be due to the very short period between the actual start of the study and filling out the questionnaires on one hand and implementation difficulties on the other. In total 7 pharmacies had either a difficult start or PCS faded into the background after a good start. These facts may have contributed to the reduced ability of learning-by-doing for pharmabuddies as almost 2/3 of included patients only interacted with the pharmabuddy once. Another plausible explanation is that the training provided to technicians and pharmacists may have been inadequate, or that technicians did not seek timely assistance from pharmacists. Furthermore, the lack of extra paid (human) resources to perform PCS in the pharmacy was a serious hurdle for continuation, as reported also in a previous UK-based study on pharmacy services [22]. Nevertheless, patients did not experience any time constraints during their contacts with pharmabuddies. This may indicate that after getting over this hurdle, PCS can be implemented in pharmacies, even without extra reimbursement on the short term, but a larger scale implementation and continuation of PCS will need extra resources to make this a success. Responding patients appreciated the personal approach, communication skills, accessibility and extra logistic support in combination with specific practicalities. Although others preferred to discuss complex PD-related problems with their PD specialists, apparently due to the knowledge gap of pharmabuddies or insufficient collaboration with other HCPs, at this point. The type of interventions by pharmabuddies was divers, but focused on pharmacotherapeutic issues and information/support consultation (e.g. switch of olanzapine to clozapine and (re-)assurance on PD medication intake with food).

This study used the Bowen’s Framework for feasibility studies to support several viewpoints on feasibility in order to shed light on the broader perspective of the PCS. The patient- and pharmacy perspectives were thoroughly included by means of questionnaires and interviews. Interviewed patients covered all PD stages, with a relative high percentage of females (67%), not reflecting the male dominance in the PD population.

### Limitations

It is likely that there was a selection bias among the patients who were included in the study and specifically those who were interviewed due to Hawthorne effects. This could have contributed to response bias. Furthermore, The collection of patient records was limited, which was largely due to the Covid-19 pandemic, but also to a lack of extra resources to perform this task. Practicality was difficult to interpret, without the inclusion of financial allocation information (e.g. time spent in the pharmacy on PCS). Participating pharmacies were mainly located in the direct vicinity of (planned) Parkinson Expertise Centers [23]. These centres collaborate already with a great number of specialised first-line HCPs, like physiotherapists, speech therapists and psychologists. This may have promoted participation and implementation of PCS in these regions. The downside of these well-organized PD expertise centers is that it may have influenced the willingness of patients to discuss problems with pharmabuddies. Again, lack of time and resources is potentially the reason for the rather limited percentage of pharmacies continuing PCS.

### Future perspectives

Our feasibility study has shown promising data on the PCS concept. However, we need clear measurable benefits from PCS, to implement this on a large scale. F.i. less adverse events, leading to a decreased number of consultations of all involved HCPs, in all healthcare settings including neurologists, specialized PD nurses, GPs. A reduced number of hospital admissions, emergency department-, outpatient clinic visits and patient reported outcome measures are important endpoints to include in future trials on this topic. Second, in order to implement PCS, improvements of the above mentioned endpoints should create financial space to reallocate extra resources in the pharmacy teams, which is needed for a sustainable, high-quality PCS concept. For improvement of collaboration with other HCPs embedding pharmabuddies into a PD Network like ParkinonNet could enhance efficacy of PCS [24, 25].

## Conclusion

This study shows PCS is appreciated by patient respondents, and could be feasible to become part of standard pharmaceutical PD care. However, the lack of knowledge on the clinical impact of PCS and several implementation issues are still present, which are mainly related to long-term effects and reimbursement. Future studies therefore should focus on these unmet needs, and should incorporate adequate endpoints to solve these issues.

## Supplementary Information

Supplementary Material 1.

Supplementary Material 2.

Supplementary Material 3.

Supplementary Material 4.

Supplementary Material 5.

## Data Availability

All data generated during and/or analysed during the current study is available and included in this published article and its supplementary information files. If someone wants to request any further information about data and materials, CCM Stuijt can be contacted: stuijt@apomed.nl.

## References

[1] Dorsey ER, Bloem BR. The Parkinson pandemic—a call to action. JAMA Neurol. 2018;75:9. 29131880 10.1001/jamaneurol.2017.3299

[2] Tenison E, Henderson EJ. Multimorbidity and frailty: tackling complexity in Parkinson’s disease. J Parkinsons Dis. 2020;10:S85-91. 32741841 10.3233/JPD-202105 PMC7592667

[3] Federatie van Medisch Specialisten. Multidisciplinaire richtlijn ziekte van Parkinson. Alphen a/d Rijn: Van Zuiden Communications; 2020. https://richtlijnendatabase.nl/richtlijn/ziekte_van_parkinson/startpagina_ziekte_van_parkinson.html.

[4] Willson MN, Greer CL, Weeks DL. Medication regimen complexity and hospital readmission for an adverse drug event. Ann Pharmacother. 2014;48:26–32. 24259639 10.1177/1060028013510898

[5] McLean G, Hindle JV, Guthrie B, Mercer SW. Co-morbidity and polypharmacy in Parkinson’s disease: insights from a large Scottish primary care database. BMC Neurol. 2017;17:126. 28666413 10.1186/s12883-017-0904-4 PMC5493890

[6] Garin N, Sole N, Lucas B, Matas L, Moras D, Rodrigo-Troyano A, et al. Drug related problems in clinical practice: a cross-sectional study on their prevalence, risk factors and associated pharmaceutical interventions. Sci Rep. 2021;11:883. 33441854 10.1038/s41598-020-80560-2 PMC7807048

[7] Yi Z-M, Willis S, Zhang Y, Liu N, Tang Q-Y, Zhai S-D. Impact of a collaborative pharmaceutical care service for patients with Parkinson’s disease. Front Pharmacol. 2022;12:793361. 35046815 10.3389/fphar.2021.793361 PMC8762333

[8] Schröder S, Martus P, Odin P, Schaefer M. Drug-related problems in Parkinson’s disease: the role of community pharmacists in primary care. Int J Clin Pharm. 2011;33:674–82. 21710194 10.1007/s11096-011-9526-x

[9] KNMP. Ministre of Health Care satified about Pharmabuddy results [Minister Bruins tevreden over resultaten Farmabuddy]. Pharmaceutisch Weekblad. 2018;153:23–23.

[10] SIR Institute for Pharmacy Practice and Policy. SIR Farmabuddy Scholingsprogramma [SIR Pharmabuddy educational program]. 2022. https://sirstevenshof.nl/product/farmabuddy-module-parkinson-8-okt-2024/. Accessed 11 Aug 2024.

[11] Bowen DJ, Kreuter M, Spring B, Cofta-Woerpel L, Linnan L, Weiner D, et al. How we design feasibility studies. Am J Prev Med. 2009;36:452–7. 19362699 10.1016/j.amepre.2009.02.002 PMC2859314

[12] Han MA, Kwon I, Reyes CE, Trejo L, Simmons J, Sarkisian C. Creating a “Wellness pathway” between health care providers and community-based organizations to improve the health of older adults. J Clin Gerontol Geriatr. 2015;6:111–4.

[13] Cené CW, Haymore LB, Ellis D, Whitaker S, Henderson S, Lin F-C, et al. Implementation of the power to prevent diabetes prevention educational curriculum into Rural African American Communities. Diabetes Educ. 2013;39:776–85. 24129595 10.1177/0145721713507114 PMC4429528

[14] van den Bemt PMLA, van der Schrieck-de Loos EM, van der Linden C, Theeuwes AMLJ, Pol AG. Effect of edication reconciliation on unintentional medication discrepancies in acute hospital admissions of elderly adults: a multicenter study. J Am Geriatr Soc. 2013;61(8):1262–8. 10.1111/jgs.12380 23869999

[15] Guy W. ECDEU Assessment Manual for Psychopharmacology. Rockville MD: US Department of Heath, Education, and Welfare Public Health Service Alcohol, Drug Abuse, and Mental Health Administration; 1976.

[16] Anja Desomer, Koen van den Heede, Mattanja Triemstra, John Paget, Dolf de Boer, Laurence Kohn, et al. Use of patient-reported outcome and experience measures in patient care and policy. Health Services Research (HSR). 2018. https://www.nivel.nl/sites/default/files/bestanden/KCE_use_of_PROM_PREM.pdf? Accessed 11 Aug 2024.

[17] National Clinical Guideline Centre (UK). Patient Experience in Adult NHS Services: Improving the Experience of Care for People Using Adult NHS Services: Patient Experience in Generic Terms. 2012. https://www.nice.org.uk/guidance/cg138. 23285499

[18] Nederlands Huisartsen Genootschap, Nederlandse Vereniging voor Klinische Geriatrie, Orde van Medisch Specialisten. Multidisciplinaire Richtlijn Polyfarmacie bij ouderen [multidisciplinary Guideline polypharmacy elderly]. 1st edition. Utrecht: NHG; 2020.

[19] Verantwoording Verkenning Farmabuddyprogramma en Ontwikkeling aanvullende scholing. [accountability exploration Pharmabuddy program and development of additional education]. Leiden; 2020.

[20] Plouvier AOA, Olde Hartman TC, Verhulst CEM, Bloem BR, van Weel C, Lagro-Janssen ALM. Parkinson’s disease: patient and general practitioner perspectives on the role of primary care. Fam Pract. 2017;34:227–33. 28419289 10.1093/fampra/cmw115

[21] Vinterflod C, Gustafsson M, Mattsson S, Gallego G. Physicians’ perspectives on clinical pharmacy services in Northern Sweden: a qualitative study. BMC Health Serv Res. 2018;18:35. 29361941 10.1186/s12913-018-2841-3 PMC5781320

[22] Ryan K, Patel N, Lau WM, Abu-Elmagd H, Stretch G, Pinney H. Pharmacists in general practice: a qualitative interview case study of stakeholders’ experiences in a West London GP federation. BMC Health Serv Res. 2018;18:234. 29609603 10.1186/s12913-018-3056-3 PMC5879559

[23] Punt voor Parkinson. Center of Excellence on Parkinson’s disease [Punt voor Parkinson]. 2022. www.puntvoorparkinson.nl. Accessed 11 Aug 2024.

[24] van de Warrenburg BP, Tiemessen M, Munneke M, Bloem BR. The architecture of contemporary care networks for rare movement disorders: leveraging the ParkinsonNet experience. Front Neurol. 2021;12:638853. 33859608 10.3389/fneur.2021.638853 PMC8042326

[25] Rose O, Happe S, Huchtemann T, Mönig C, Ohms M, Schwalbe O, et al. Enhancing medication therapy in Parkinson’s disease by establishing an interprofessional network including pharmacists. Int J Clin Pharm. 2021;43:441–8. 33893597 10.1007/s11096-021-01263-w

