# Supplementary material for: Feasibility of a Pharmabuddy Care Service for patients with Parkinson’s disease
Source: BMC Health Serv Res. 2024 Dec 18;24:1560. doi: 10.1186/s12913-024-12057-x (PMC11654004; doi:10.1186/s12913-024-12057-x)
Supplement: Supplementary file 1 — Supplementary Material 1. [file 12913_2024_12057_MOESM1_ESM.docx]

## Appendix 1 PCS education

*Trainers*

Pharmacy teams consisting of one or two pharmacy technicians and a pharmacist, were trained by three PD specialized and experienced healthcare professionals: a Pharmabuddy (pharmacy technician), - nurse and - pharmacist. At the start of the first course, the trainers were all accredited by the national movement disorder network ParkinsonNet for respectively 3, 4 and 6 years[23]. The structure of the course was to start with the implementation of the PCS shortly after the first course-day.

*Content of training*

Education consisted of three group sessions over a one year period (one day physical education for couples pharmacy technician(s) and pharmacists, after 2 months one online session for practical issues for pharmabuddies and one on specialized medication review for pharmacists. Furthermore, pathophysiology, pharmacology and practical issues e.g. on getting knowledge of a patient’s preference and wishes, other involved healthcare professionals and how to get in contact with them in case a problem cannot be solved in the pharmacy, was covered. They were provided with materials to support implementation and execution. These comprised an intake form with accompanying FAQs, information on frequent medication- an disease related problems for PD patients, format for a project plan on implementation and an information letter for GP/homecare.

*Implementation*

Implementation and management of the PCS in the pharmacy as well as intervision (to share their experiences with the group in order to improve implementation/management), was covered. It was advised to inform involved stakeholders e.g. GPs, homecare, neurologists and/or Parkinson nurse on the intention to start with a structured approach of PD patients.

Before each session, pharmacy teams had to prepare several topics like an intake/interview with a PD patient and preparation of an implementation plan of the PCS. The total program was accredited for pharmacy technicians and pharmacists with resp 6,5 and 8,5 hours.
