# Supplementary material for: Feasibility of a Pharmabuddy Care Service for patients with Parkinson’s disease
Source: BMC Health Serv Res. 2024 Dec 18;24:1560. doi: 10.1186/s12913-024-12057-x (PMC11654004; doi:10.1186/s12913-024-12057-x)
Supplement: Supplementary file 2 — Supplementary Material 2. [file 12913_2024_12057_MOESM2_ESM.docx]

## Appendix 2 Patient questionnaire

A questionnaire was drafted for patients consisting of 15 questions and was made available on paper and electronically in Survalyzer. The questionnaire was tested by a patient with Parkinson’s disease on comprehensibility and duration on filling out, not leading to major changes [15]. Pharmabuddies were asked to invite all patients to whom they were providing PCS and had been contacted at least one time, to participate. Patients who were able and willing to participate received the questionnaire. Due to the personal approach before sending the questionnaire, reminders were unnecessary.

Within the first domain *acceptability* suboutcome *satisfaction rating (1.1.1)* of the pharmabuddy was questioned quantitatively on a scale of 0-10 (Q 12). The *experienced PCS* *(1.1.2)* was obtained by eight Likert-scale questions based on the Dutch Patient Reported Experience Measures (PREM) chronic care with a focus on communication and knowledge (Q 6) (15)[15]. Bowen’s domains *demand* and suboutcome *Contact frequency in the last 6 months* *(2.1.1)* was questioned with a multiple choice question (Q 2) and *perceived benefits or deterioration (4.1.1)* were obtained from open-ended questions about the added value of the PCS to the patient with a focus on change in (PD) complaints(Q 7, 8). *Facilitators and barriers/tips and tops for implementation from patient perspective* (*3.2.2*) were extracted from Q11 and 12. To measure *limited efficacy* specifically *the effect on PD symptoms (4.1.2)*, a change in complaints was measured as a single measurement adapted from the Patient Global Impression of Change[23].

1. You as a patient with Parkinson’s disease was provided with a fixed contact person in your pharmacy. This can be a pharmacy technician or a pharmacist and they are called a pharmabuddy. Is it correct that you have such a contact person in your pharmacy?

Yes

No. In case you ticked this box, you don’t have to continue with the questionnaire. Thank you very much for your cooperation.

Don’t know . In case you ticked this box, you don’t have to continue with the questionnaire. Thank you very much for your cooperation.

1. How often have you contacted the pharmabuddy in the last six months?

No contact at all. 🡪 you are referred to question 3.

1 to 2 times. 🡪 you are referred to question 5.

3 times or more. 🡪 you are referred to question 5.

1. Which topics have been discussed with the Pharmabuddy? E.g. medication intake, handling, constipation, personal situation. In case you can’t remember any subject anymore you can tick the box ‘don’t remember’. _________________________________________________________________­­­­­­________

_________________________________________________________________________

_________________________________________________________________________

_________________________________________________________________________

don’t remember.

1. A few subjects are included below. They pertain to every interaction you have ever had with a Pharmabuddy. How frequently they are relevant. Please check the box that best matches.

| **Topic** | **Always** | **often** | **Sometimes** | **never** | **NA** |
| --- | --- | --- | --- | --- | --- |
| Listening |  |  |  |  |  |
| Possibility to ask questions |  |  |  |  |  |
| Interested in personal situation |  |  |  |  |  |
| Advice for personal situation |  |  |  |  |  |
| Good explanation |  |  |  |  |  |
| Knowledge on PD treatment |  |  |  |  |  |
| Addition on top of other HCPs |  |  |  |  |  |
| Brand switch |  |  |  |  |  |
|  |  |  |  |  |  |

1. Did the interaction with the specialized pharmacy technician, pharmabuddy bring you anything?

yes 🡪 go to question 7.

No. 🡪 go to question 10.

1. What did this interaction produce for you? __________________________________________________________________­­­­­­________

__________________________________________________________________________

__________________________________________________________________­­­­­­________

1. Parkinson’s disease has many faces, it can present with a wide spectrum of complaints. We would like to know whether your complaints have been improved since you have a Pharmabuddy?

No

don’t know.

yes, the next complaints have been improved:

_____________________________________________________________________

_____________________________________________________________________

_____________________________________________________________________

1. Or have the complaints deteriorated?

No

Don’t know.

yes, the next complaints have been deteriorated:

_____________________________________________________________________

_____________________________________________________________________

_____________________________________________________________________

1. Which manner has PCS contributed to improvement or deterioration of these complaints?

If you don’t know, please tick the box below.

_______________________________________________________________­­­­­­_________

___________________________________________________________________________

__________________________________________________________________­­­­­­_________

___________________________________________________________________________

Don’t know.

1. Would you recommend a Pharmabuddy to other patients with Parkinson’s disease? A 0 means not at all, a 10 you would highly recommend the Pharmabuddy.

| not at all |  |  |  |  |  |  |  |  |  | for sure |
| --- | --- | --- | --- | --- | --- | --- | --- | --- | --- | --- |
|  |  |  |  |  |  |  |  |  |  |  |
| 0 | 1 | 2 | 3 | 4 | 5 | 6 | 7 | 8 | 9 | 10 |

1. Would you like to give the pharmabuddy a compliment? If so, what type of feed-back would you like to give to the pharmabuddy?

_____________________________________________________________­­­­­­________

_____________________________________________________________________

_____________________________________________________________­­­­­­________

1. What could your pharmabuddy have done better?

_____________________________________________________________­­­­­­________

_____________________________________________________________________

_____________________________________________________________­­­­­­________

_____________________________________________________________________

1. Are you willing to participate in an in-depth interview about Pharmabuddy Care Service? If so, could you please state your phone number below. (this will only be used for the interview fort his investigation and nothing else and will be discarded afterwards.

yes, my phone number is: __________________________________

no, I will not participate.
