# Supplementary material for: Feasibility of a Pharmabuddy Care Service for patients with Parkinson’s disease
Source: BMC Health Serv Res. 2024 Dec 18;24:1560. doi: 10.1186/s12913-024-12057-x (PMC11654004; doi:10.1186/s12913-024-12057-x)
Supplement: Supplementary file 3 — Supplementary Material 3. [file 12913_2024_12057_MOESM3_ESM.docx]

## Appendix 3a Patient interview guide

A topic guide for the interviews was developed in order to collect information on the Bowen domains acceptability and implementation/practicality. *Experienced PCS (1.1.2), Facilitators and barriers/ tips and tops for implementation from patient perspective (3.2.2)* was derived by these questions.

To obtain information on the *expressed interest or intention to use* *(2.2.1*), patients were asked about the ability to participate in their own care while supported by the pharmabuddy topics ‘enable patients to actively participate in their care’ and ‘essential requirements of care’). *Perceived benefits (4.1.1)* and *effect on PD symptoms (4.1.2)* explicitly were questioned by any change in complaints and the effect on overall wellbeing.

In developing the question, the by the National Health Service described ‘themes for patient experience recommendations’ were taken into account(6)

After two interviews the topic guide was tested for adjustments. No adjustments were needed, therefore all interviews were included.

**Topic list**

| **General introduction**  What is your experience with a fixed contactperson/ a pharmabuddy in your community pharmacy?   - Continu questioning on why he/she found it good/bad/positive/negative - What was important for you? - How did you experience being provided by the pharmacy of a fixed contact person? | **Bowen framework**  **experienced PCS and tips and tops** |
| --- | --- |
| **How do you live (domestic situation) and how do you handle medication within potential constrains (period before fixed contact person)**  Domestic situation   - Living together/alone with or without homecare   Medication management   - Do you manage your medication yourself/are you able to take your medication yourself? - Do you have/need assistance for medication intake?   Are you satisfied with medication:   - In general, experiences/ satisfaction (positive/negative/neutral) - Current situation around your disease - Adverse drug reactions/events - Own expectations/confidence in pharmacy activities   Do you think a pharmabuddy can help you with this? How? | **Knowing the patient as an individual and how do they handle their medication, what are their needs? How does PCS fit in with this?** |
| **Supplied PCS and effects**  Contact with pharmacy staff   - How often ? - With whom? - By whom initiated?   You stated in the questionnaire your complaints have changed.   - Can you explain more about this change, these complaints? - In case of change in complaints: was it your own feeling or objectivated by a HCP? - Effects on general well-being? | **contact frequencies, who’s initiative?**  **Effect on (PD) symptoms, well beiing** |
| **Experiences with PCS/the pharmabuddy ?**  Has anything (been) changed for you ?   - Regarding the pharmacy interaction or individual circumstances - Would you prefer to have a larger say in the decisions that are made? Do you think you have the ability to co-decide with others? - Shared-decision making   In your communication with the pharmabuddy, did you miss anything?  How did you experience the contact?   - Hospitality/ helpful/ accessible - Have you felt assisted? - Did you anticipate receiving assistance or contact moments? | **Enable patients to actively participate in their care**  **experienced PCS**  **expectations/yield** |
| **Involved healthcare professionals**  Which other HCPs are involved in your treatment except the GP?  Do you have any additional illnesses that could be causing further HCP involvement??  How well are the HCPs working together and coordinating?  Has pharmabuddy to offer unique features as compared to other HCPs? | **Continuity of care and relationships** |
| **Completion**  Would you like to participate in other investigations? |  |

[NHS 2012;Plochg 2007; Thorogood 2014]

## Appendix 3b Patient records

Data was collected on description of the topic of the contact moment, by whom it was initiated (*demand (2.1.1*), what type of question by whom or what medication-related problem was found. Furthermore, actions, advices and/or interventions executed on these questions or problems (*limited-efficacy 4.2.1 and 2*) were collected.
