# Supplementary material for: Feasibility of a Pharmabuddy Care Service for patients with Parkinson’s disease
Source: BMC Health Serv Res. 2024 Dec 18;24:1560. doi: 10.1186/s12913-024-12057-x (PMC11654004; doi:10.1186/s12913-024-12057-x)
Supplement: Supplementary file 4 — Supplementary Material 4. [file 12913_2024_12057_MOESM4_ESM.docx]

## Appendix 4 Pharmabuddy questionnaire

The final questionnaire comprised 17 questions which covered Bowen’s domains *acceptability (1.2), demand (2.3, 2.4), implementation/practicality (3.2.,3 and 4))* on twelve sub-outcomes. Questions were on implementation and the extent of it (numbers of pharmabuddies, patients), way of implementation (patient selection, information and coordination with other HCPs), facilitators and barriers for implementations, experience with the education and PCS overall.

For *the opinion of pharmabuddy about the service* *(1.2.1) (Q8+9)* an open-ended question on experience was included. *Number of patients in PCS program* *(2.3.1) (Q4), lag time between course and intervention initiation* *(3.2.3)(Q5)* and *stakeholder involvement* *(3.2.4) (Q2,14)* all were questioned with multiple choice options. *PCS providing pharmacies* *(2.3.2),* *active pharmabuddies per pharmacy* *(2.3.3)(Q3)* and *which type of HCPs referred to PCS (2.4.1)(Q6, 14)* was questioned by the absolute number or type followed by an explanation option. *Use of supportive materials* *(**3.2.1)* (Q12+13) was questioned by presenting all provided materials on a Likert scale for use. Agreement on implementation effort by a close-ended question (Q15) was followed by explanation options (Q16,17) (*extent of implementation effort* *(3.2.2))*. Potential barriers and facilitators for implementation were questioned by presenting several topics on a Likert scale for degree of (dis)agreement *(facilitators and barriers for implementation* *(3.3.1)*) and numbers of *pharmacies started inviting patients* *(3.4.2*) (Q7). The *way of patient identification* (*3.4.1*) (Q1), *perceived presence of necessary knowledge* (3.3.1)(Q 10,11)) was questioned by a multiple choice followed by an open-ended question.

Q1:What actions have been done to find patients with Parkinson’s disease eligible for PCS?

*Numerous responses are possible!*

Selection of people using the prescribed drugs listed below: ______________________________

prescription for the following drugs: ______________________________

repeated discharge prescriptions .

Prior to initiating the unit dosage packaging according to details from another conversation with the patient or the caregiver.

Another thing, this is:______________________________

No internal appointments have been made regarding this subject.

Q2 Which other healthcare professionals have you informed about the provision of PCS to Parkinson's disease patients?

*Numerous responses are possible!*

No other HCPs have been informed

General practitioners

homecare

specialised GP

Consultationteam in the hospital

Specialized PD nurse

neurologist

other, namely: ______________________________

The following inquiries relate to the Pharmabuddy care service that have actually been provided to PD patients up to this point.

Q3a: How many Pharmabuddies Parkinson are currently active in your pharmacy?

*This implies that they are available to provide the care for patients the moment they present.*

*______________________________(validation: number)*

Q3b: Indien het niet goed aan te geven is kunt u hieronder uw antwoord toelichten.

*______________________________*

Q4: Hoeveel patiënten met de ziekte van Parkinson hebben tot nu toe naar schatting **in totaal** een buddy of buddykoppel toegewezen gekregen? Een patiënt telt ook mee als hij/zij een buddy toegewezen gekregen had, maar al overleden was voordat de eerste zorg geleverd kon worden.

0 patients

1 to 4 patients

5 to 14 patients

15 to 24 patients

25 or more patients

Q5: How much time passed after the 3-day course ended before the first PD patients were linked up with a Pharmabuddy?

Prior to the course, we provided palliative care patients with PCS.

Less than 2 months

Between 2 and 6 months

Between 6 and 12 months

more than a year

Q6: Which healthcare professionals have referred Parkinson's disease patients for PCS?

*Numerous responses are possible!*

Non

General practitioners

homecare

specialised GP

Consultationteam in the hospital

Specialized PD nurse

neurologist

other, namely: ______________________________

Q7:Which PCS scenario best describes it in your pharmacy, in your opinion?

*Only one response is possible; further explanation is allowed in the following question.*

It didn't really have a priority at first.

We had a great beginning, but we never truly delivered PCS.

Following a promising beginning and the delivery of care to a few people with Parkinson's disease, it faded into the background.

Although we had a challenging beginning, we are now providing Parkinson's disease patients with PCS.

We began immediately following the training and are still providing PCS.

Other namely: ______________________________

Q8: Please provide more details in your response, such as why PCS was not prioritised, why it never truly got started, or why you had a successful start but the actual PCS provision is no longer available.

*______________________________*

Q9: Please elaborate in your response, mentioning the reasons behind your challenging beginnings and the steps you took to successfully provide PCS to patients with Parkinson's disease.

*______________________________*

The following inquiries concern the Pharmabuddy course.

Q10: Do you believe you had sufficient **knowledge** to begin PCS Parkinson?

Yes

No, I missed knowledge on : ______________________________

Q11: Do you believe you had sufficient **skills** to begin PCS Parkinson?

Yes

No, I missed the skills on : ______________________________

Q12:Are you aware of the following documents that were given to you during the Pharmabuddy Parkinson course, and have you made use of them?

|  | No, don’t know | Yes, i am aware but we didn’t use it | Yes, I’m aware and we used it | Don’t remember |
| --- | --- | --- | --- | --- |
| information leaflet |  |  |  |  |
| information letter GP, homecare |  |  |  |  |
| Intake form |  |  |  |  |
| Checklist physical condition |  |  |  |  |
| evaluation form |  |  |  |  |
| Holiday letter |  |  |  |  |
| Instructions for pharmacy information computersystems (in Pharmacom and Mira) |  |  |  |  |
| Starting and shaping the process: some advice |  |  |  |  |

Q13: Would you have preferred more samples of materials or documents?

yes, namely: ______________________________

No

Q14: collaboration with other HCPs concerning PCS
How have you approached other HCPs for referrals?

*numerous responses are possible!*

Prior to PCS commencing, we had already referrals.

We gave out a project pamphlet.

We individually notified HCPs when PCS began..

During the Pharmacotherapeutic Consultation (FTO), it was discussed (local regular GP, pharmacist consultation)

Other, namely: ______________________________

Q15: Do you believe that your post-course efforts were sufficient to begin PCS?

Yes

No

Q16: Who was the more responsible party?

the pharmacist

Me and an additional pharmabuddy

someone else namely: ______________________________

Q17: What else might have been done?
