# Supplementary material for: Feasibility of a Pharmabuddy Care Service for patients with Parkinson’s disease
Source: BMC Health Serv Res. 2024 Dec 18;24:1560. doi: 10.1186/s12913-024-12057-x (PMC11654004; doi:10.1186/s12913-024-12057-x)
Supplement: Supplementary file 5 — Supplementary Material 5. [file 12913_2024_12057_MOESM5_ESM.docx]

## Outcome Appendix Table 1 Quantitative results for Pharmabuddy Care Service on Parkinson’s disease from patient and pharmabuddy perspective

| **Domain** | **Outcome**  **(from questionnaire)** | **Results** |
| --- | --- | --- |
| **1. Acceptability** | 1.1 Patient satisfaction and appreciation of PCS (n=23) | An average 9.5 (SD 1.3) out of 10 (recommendation pharmabuddies to other PD patients by rating their satisfaction with PCS) |
|  | 1.2 situation around PCS in pharmacy according to Pharmabuddies (n=12) | 5 good at the start, disappeared after time  4 a good start and were still active  2 had a difficult start but are now active with PCS  1 started but never delivered PCS |
| **2. Demand** | 2. 1 Actual use by patients  (n=36)  Contact initiated by patients (n=21 patients^*^) | 23 (59%) patients filled the questionnaire  1 contact moment (in the preceding 6 months): 15 (42%) patients  1-2 contact moments: 5 (14%) patients  3 or more contact moments: 10 (28%) patients  six (17%) did not remember the contact moment |
|  |  | 8 (38%) patients with 18 (1-7 per person) contact moments. |
|  | 2.3 Actual PCS provision by pharmabuddies (n=12) | Five pharmacies: 1-4 patients, five pharmacies: 5 -14 patients one had 15 to 24 patients, One pharmacy: zero.  In total: 11 pharmacies provided PCS |
|  |  | 8 pharmacies: 1-2 pharmabuddies  2 had one pharmabuddy, 1 had no pharmabuddy  1 pharmacy stated to have 30 buddies: all pharmacy technicians were appointed as pharmabuddy |
|  | 2.4 Referrals according to pharmabuddies (n=12) | 4 pharmacies : by Parkinson nurses  1 pharmacy: GP  1 pharmacy: homecare  (Before the start of PCS none of participating pharmacies had referrals from other HCPs) |
| **3. Implementation/ Practicality** | 3.2 Indicators of implementation according to pharmabuddies (n=12) | 7 (58%) pharmacies : intake form  3 (25%) pharmacies : information letter for GP/homecare  6 (50%) pharmacies: used tips to start  2 (17%) pharmacies lacked an information letter for the patient |
|  |  | sufficient effort to start with PCS : 7 (58%)  either pharmacists or other pharmabuddies should have done more: 2 (17%)  1 (8%) would have liked to have more support from the pharmacist |
|  |  | 7 (58%) pharmacies: already active with PCS palliative care, amplified this to PD patients (three did not mention the timeframe of their start)  7 (58%) started within 2 months  2 (17%) pharmacies: 2-6 months |
|  | 3.3 Barriers and facilitators according to pharmabuddies (n=12) | information of other HCPs: via FTO (7 GPs) or face to face (4)  8 pharmacies approached GPs  8 specialized Parkinson nurse  3 the neurologist  2 homecare  1 pharmacy: non |
|  |  | For 6 (50%) pharmabuddies a strong facilitator: positive reactions of patients and their proxies  7 (58%) facilitator (to some extent): GPs and homecare giving positive reactions (one mentioned both subjects) |
|  |  | 11 (92%) pharmabuddies judged their knowledge on PD sufficient to start with PCS  1 needed more basics and simple explanation of information  All pharmabuddies were sufficiently skilled after the course |
|  |  | tops: very positive on listening also for proxies  implementation of knowledge in advices  Tips: 2 patients advised to organise information evenings for PD patients and their family, others to pay more attention to the proxies and non- native patients with PD.  Knowledge on the disease could be amplified |
|  | 3.4 Conditions for PCS according to pharmabuddies (n=12) | 8 (44%) pharmacies made a selection on PD medication   1. Identified patients by prescriptions with PD medication 2. At the introduction of an unit dose packaging 3. based on a selection of contraindication PD 4. no specific approach for patients   Constraints mentioned were:   - time (n=10, 83%) influenced strongly the start and performance (eg absence of colleagues and difficulties with handing over work in case the pharmabuddy had to act upon one of their patients) - Absence of enthusiasm of the whole team - inability to get in contact with PD patients (one pharmabuddy) - a patient preferred other HCPs above pharmabuddy contact (one pharmabuddy) |
|  |  | Six (50%) (2 of those with a late start) had a good start and still were active  five (42%) stated PCS activities faded into the background after a good start  one did not start |
| **4. Limited efficacy** | 4.1 Clinical outcomes according to patients (n=23) | 20 (87%) patients had a positive effect induced by the contact with a pharmabuddy on:  clarification on medication intake, knowledge on Parkinson’s disease, explanation on the role of the pharmacy and other HCPs, reassurance and (partial) solution of complaints  Patients without any effect from the contact with a pharmabuddy expressed no reasons for this fact.  One patient declared a deterioration (cramps, less endurance)  One: not possible to answer the question on deterioration or improvement  all others (10, 23%) had no change in complaints |
|  |  | 6 (26%) patients: improvement of one or more complaints (on state of mind, motor symptoms (4), gastrointestinal symptoms and effectiveness of levodopa)  10 (44%) : neutral  7 (30%) no change of complaints  One patient mentioned a worsening of condition (see above) |
|  | 4.2 medication-related problems + interventions  (n=21 patients^*^) | 21 patients (mean age 68 SD ± 7,3) had 93 (median 3 (range 1-16) problems/questions per patient |
|  |  | 89 interventions on 72 problems in the category ‘clinical’ (see appendix 5). This comprised (advices for) changes in medication, information and advice.  22 pharmacotherapeutic proposed interventions: 59% accepted, 41% was not documented or unknown |

^*^ from patient records

FTO: PharmacoTherapeutic Consultation between pharmacists and GPs
